# Diffusion MRI correlation with p16 status and prediction for tumor progression in locally advanced head and neck cancer

**DOI:** 10.3389/fonc.2023.998186

**Published:** 2023-12-21

**Authors:** Yue Cao, M. Aryal, P. Li, C. Lee, M. Schipper, D. You, E. Jaworski, L. Gharzai, J. Shah, A. Eisbruch, Michelle Mierzwa

**Affiliations:** ^1^ Departments of Radiation Oncology, University of Michigan, Ann Arbor, MI, United States; ^2^ Department of Radiology, University of Michigan, Ann Arbor, MI, United States; ^3^ Department of Biomedical Engineering, University of Michigan, Ann Arbor, MI, United States; ^4^ Department of Biostatistics, University of Michigan, Ann Arbor, MI, United States; ^5^ Department of Radiation Oncology, VA Ann Arbor Healthcare System, Ann Arbor, MI, United States

**Keywords:** diffusion, magnetic resonance imaging, HNSCC, p16+, imaging biomarker

## Abstract

**Purpose:**

To investigate p16 effects on diffusion image metrics and associations with tumor progression in patients with locally advanced head and neck cancers.

**Methods:**

Diffusion images pretreatment and after 20 Gy (2wk) of RT were analyzed in patients with cT4/N3 p16+ oropharynx cancer (OPSCC) (N=51) and locoregionally advanced head and neck squamous cell carcinoma (LAHNSCC) (N=28), enrolled onto a prospective adaptive RT trial. Mean ADC values, subvolumes with ADC <1.2 um^2^/ms (TV_LADC_), and peak values of low (µ_L_) and high (µ_H_) components of ADC histograms in primary and total nodal gross tumor volumes were analyzed for prediction of freedom from local, distant, or any progression (FFLP, FFDP or FFLRDP) using multivariate Cox proportional-hazards model with clinical factors. P value with false discovery control <0.05 was considered as significant.

**Results:**

With a mean follow up of 36 months, 18 of LAHNSCC patients and 16 of p16+ OPSCC patients had progression. After adjusting for p16, small µ_L_ and ADC values, and large TV_LADC_ of primary tumors pre-RT were significantly associated with superior FFLRDP, FFLP and FFDP in the LAHNSCC (p<0.05), but no diffusion metrics were significant in p16+ oropharynx cancers. Post *ad hoc* analysis of the p16+ OPSCC only showed that large TV_LADC_ of the total nodal burden pre-RT was significantly associated with inferior FFDP (p=0.05).

**Conclusion:**

ADC metrics were associated with different progression patterns in the LAHNSCC and p16+ OPSCC, possibly explained by differences in cancer biology and morphology. A deep understanding of ADC metrics is warranted to establish imaging biomarkers for adaptive RT in HNSCC.

## Introduction

Diffusion magnetic resonance imaging (MRI) measures water mobility in the tissue environment with high sensitivity to microstructures of cells and cell membrane permeability. Apparent diffusion coefficient (ADC), a commonly used diffusion imaging parameter, has been shown to be prognostic and predictive for outcomes in head and neck squamous cell carcinomas (HNSCC) (1–7). Diffusion MRI does not require a gadolinium (Gd) based contrast agent and can be obtained within a few minutes. The simplicity of ADC from acquisition to computation as well as its predictive value results in advantages of ADC as an emerging imaging biomarker for stratifying progression risk in patients with HNSCC during adaptive (de-escalation or intensification) radiation therapy (RT).

Recent studies have shown that pre-treatment ADC values in p16- HNSCC tumors are greater than in p16+ ones, although with varying levels of significance (4, 8–10). It is known that p16+ oropharynx cancers have improved outcomes compared to other locally advanced head and neck cancers (LAHNSCC) (11–13). To date, whether the prognostic or predictive value of ADC is affected by tumor biology and morphology differences between p16- and p16+ HNSCC has not been assessed. Furthermore, it has been shown that the ADC distribution in HNSCC is deviated from a Gaussian distribution, which motivates studies of skewness and kurtosis of ADC distributions (5, 9). Furthermore, the ADC distribution in HNSCC, its response to RT, and its association with progression have not been characterized to account for p16 status differences.

In this study, we aimed to quantitatively characterize the distribution of ADC and its changes during RT in poor prognosis locally advanced HNSCCs, including cT4 or N3 p16+ oropharyngeal squamous cell carcinoma (OPSCC) and LAHNSCC (composed of p16- disease and p16+ non-oropharyngeal sites). We compared parameters of ADC distributions and their association with progression patterns between p16+ OPSCC and LAHNSCC tumors. This investigation could provide insight in selection of ADC metrics for prediction of progression risk for locally advanced HNSCC.

## Methods

### Patients

Patients with locally advanced HNSCC who were enrolled in a randomized phase II clinical trial between March 2014 and January 2020 were included in this analysis (NCT02031250). This trial was approved by the Institutional Review Board of the University of Michigan. Written consent was obtained from all enrolled patients. The clinical trial results are reported elsewhere (14). In brief, eligibility included patients with 1) cT4/N3 (AJCC 8 stage III) p16+ OPSCC, 2) locally advanced (T3-4/N2-3) p16- oropharyngeal or p16+ non-oropharyngeal head and neck cancer planned to undergo definitive chemoradiation therapy (CRT). p16 status was evaluated by immunohistochemistry. The patients were randomized to a standard arm of RT (70 Gy in 35 fractions) or an experimental RT boost arm, both with concurrent weekly cisplatin (40mg/m2) or carboplatin (AUC=2) for cisplatin ineligibility. In the experimental arm, a union of 1) the persisting low blood volume (BV) [BV<7.64 ml/100g based upon a previous histogram analysis (15)] pre-RT to after 20 Gy and 2) persisting low ADC [ADC< 1.2 um^2^/ms based upon a histogram analysis of previous works (16, 17)] pre-RT to after 20 Gy received 2.5 Gy per fraction for the last 15 of 35 fractions for a total dose of 80 Gy in 35 fractions. If the union of persisting subvolumes pre-RT to after 20 Gy was less than 1 cm^3^, the patient was entered into an observation arm and treated by standard RT (70 Gy in 35 fractions).

### Diffusion imaging acquisition

Patients underwent MRI scans pre-treatment (< 2 weeks prior to the initiation of definitive CRT) and at fraction 10 (20 Gy) per protocol. All diffusion weighted (DW) images as well as T2- weighted and post-Gd T1-weighted images were acquired on a 3T scanner (Skyra, Siemens Healthineers). All patients were scanned in the treatment position using an individual-patient immobilization 5-point mask and bite block or Aquaplast mold as required for treatment. DW images were acquired by either a 2D spin-echo single shot echo-planar pulse sequence or a readout segmentation of long variable echo-trains (RESOLVE) pulse sequence that reduced geometric distortion (18) with spatial resolution of ~1.2×1.2×4.8 mm and b-values of 50 and 800 s/mm^2^. ADC maps were calculated from the two b-value DW images to mitigate the perfusion effect by using in-house software that was technically validated in a QIN collaborative project (19). Quality and geometric alignment of ADC maps were assessed and reported previously (16).

### Diffusion image metrics

Quantitative diffusion image metrics were calculated in the gross tumor volumes (GTVs) contoured manually on post-Gd T1-weighted images by the treating attending HN radiation oncologist and reviewed by the trial principal investigator (MM). Each tumor including primary and treated nodal tumors was contoured individually. Considering the dramatic reduction of gross head and neck movement during scanning by individual-patient immobilization devices, ADC maps were reformatted to match voxel-by-voxel of post-Gd T1-weighted images and overlaid with the GTV. Gross necrosis regions in the GTV were excluded by thresholding ADC below 2.7 um^2^/ms that was 10% below the value of free water diffusion.

The mean ADC value and the subvolume of low ADC thresholded at 1.2 um^2^/ms in each GTV (TV_LADC_) were calculated. Also, as a bimodal distribution of ADC values in the primary GTV was observed, suggesting two major populations, the histogram of ADC was approximated by two Gaussian functions and fitted after binned with a size of 0.1 um^2^/ms using a Simplex optimizer written in C++ (see **Supplementary Figure 1A**). The low and high ADC components in the GTV, referred as respective L and H, were described by their peak ADC values (µ), widths (σ), and amplitudes (A). If a single Gaussian-like distribution was observed, the single peak ADC component was considered as 50% of each to be the low and high components.

### Statistical analysis

The p16 effects on diffusion imaging metrics and association with tumor progression were tested using Kruskal-Wallis test and Cox proportional-hazards model. We considered freedom from local progression (FFLP), freedom from locoregional progression (FFLRP), freedom from distant progression (FFDP), and freedom from locoregional and distant progression (FFLRDP). The time to progression was defined from the starting date of RT to the date of local, regional or distant progression, and censored at other progressions that were not targeted in the test, death, or last follow-up. As approximately 95% of patients had T4/N3 diseases, p16 status and RT boost as clinical factors were considered in analysis. Smoking status as a controversial clinical factor for predicting specific patterns of progression was not included to limit overfitting (20–22). Multivariate Cox model was used to assess the image metrics one at a time with clinical factors for prediction of tumor progression. Considering multiple comparisons in the analysis, p values were corrected with false discovery rate control (FDC). The adjusted p value with FDC <0.05 was considered as significant. All analyses are summarized in **Supplementary Figure 2**.

## Results

### Patients and outcomes

We examined the imaging characteristics of 79 patients (median age of 64 years, 51 p16+ OPSCC and 8 females) randomized in an adaptive RT boost trial, 40 on the standard arm and 39 on the experimental arm. The patient characteristics are provided in **Table 1**. The details of the trial and outcomes were provided elsewhere (14). In brief, the mean follow-up was 36 months, median 30 months (range 8-83 months) for patients without death, with minimum of 12 months follow-up in all patients without disease progression except one who was lost in follow-up. LAHNSCC disease included 50% p16- oropharynx, 11% larynx, 18% hypopharynx, 14% sinonasal, and 7% EBV negative nasopharynx, with known similar outcomes (23, 24). In the 28 LAHNSCC patients, 10 had no evidence of disease (NED), 11 patients had local failure (LF) in which 4 had regional failure (RF) and 7 had distant failure (DF) at the same time, 6 had DF only, and 1 had both RF and DF. Also, 9 of the 28 received 80 Gy RT adaptive boost. In 51 of the p16+ oropharynx patients, 34 had NED in which 13 received 70 Gy standard treatment (NED_70_) and 21 had 80 Gy RT boost (NED_80_), 7 had LF in which 1 had RF at the same time, 9 had DF in which 1 had RF at the same time, and 1 had RF only. There was no effect of RT boost on FFLRP in the LAHNSCC patients and an observable boost effect in the p16+ oropharynx patients, see **Supplementary Figure 1B**, suggesting that the effect of the boosting dose may depend upon p16 status.

**Table 1:** Patient characteristics.

N	79
Age (years)	
Median (range)	64(47-79)
Sex	
F/M	8/71
Clinical prognostic group	
p16+oropharynx/LAHNSCC	51/28
T and N stages (p16+ oropharynx/LAHNSCC)	
T4N2b-3	22/15
T4N1-2a	20/3
T4N0	2/5
T3N2b-3	3/3
T3N0-2a	2/1
T0-2N3	2/0
T2N1	0/1
Radiation Dose	
80Gy/70 Gy	39/40
Chemotherapy	
Cisplatin/Carboplatin	33/46
Smoking status	
Never/Ever (‗10 pk yr)	18/61
Primary Gross Tumor Volume (cm^3^) (median(range))	
p16+ oropharynx	48.3(9.1-595.2)
LAHNSCC	62.5(30.0-280.1)
Nodal Gross Tumor Volume (cm^3^) (median(range))	
p16+ oropharynx	20.5(0.7-145.3)
LAHNSCC	20.9(2.9-274.1)
Progression, N (p16+ oropharynx/LAHNSCC)	
Total progression	34(16/18)
local	17(6/11)
regional	9(3/6)
distant	23(9/14)
death	24(8/16)

### Characteristics of imaging metrics of LAHNSCC and p16+ oropharynx cancers

Considering differences of tumor biology, outcome, and response to radiation boosting between the p16+ OPSCC and the LAHNSCC, diffusion metrics (namely TV_LADC,_ mean ADC and peak ADC values of low (µ_L_) and high (µ_H_) components) were characterized first.

**Figure 1:**
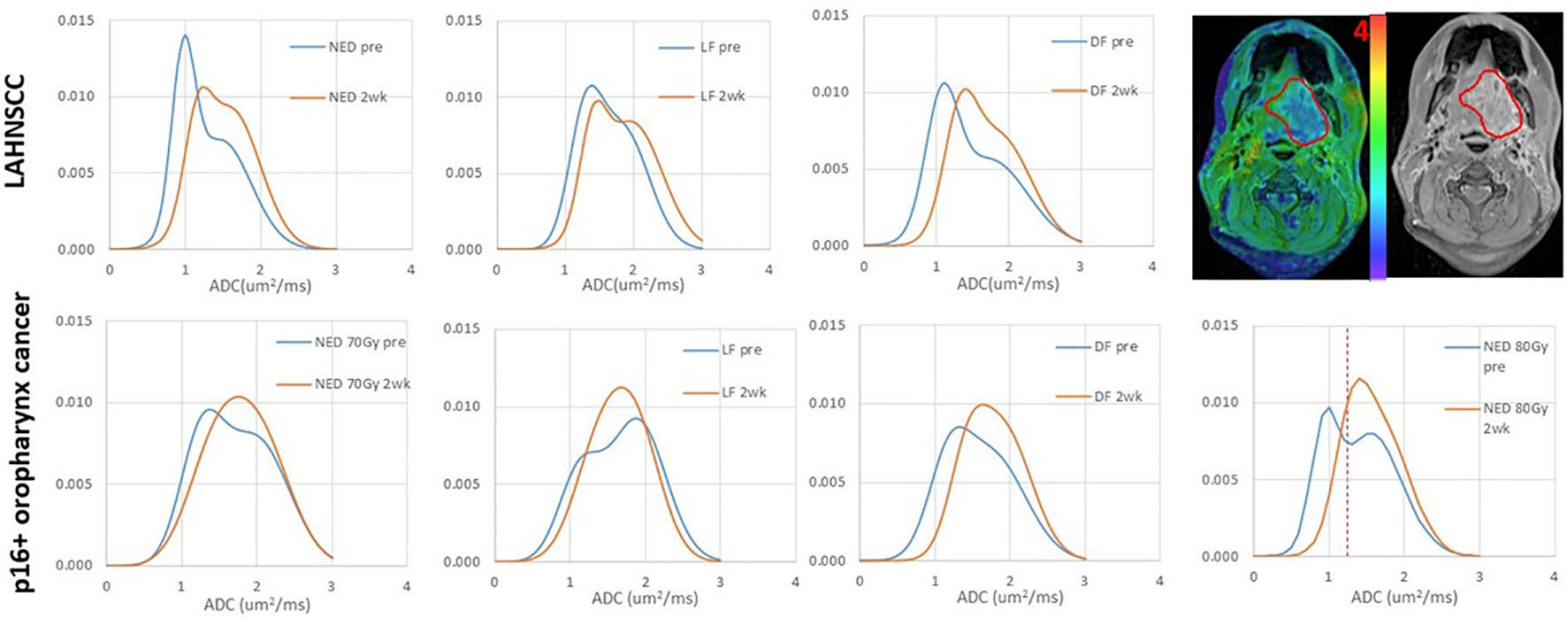
Averaged ADC histograms of the subgroups of LAHNSCC tumors (top row) and p16+ oropharynx cancers (bottom row). LAHNSCC from left to right: no evidence of disease (NED), local failure (LF) with or without regional failure (RF) or distant failure (DF), and DF only. p16+ oropharynx cancers from left to right: NED with 70 Gy RT, LF, DF, and NED with 80 Gy RT. Blue: pre-RT; orange: 2 weeks during RT. The panel at the right top corner shows an example of an ADC map overlaid on the post-Gd T1-weighted image with a gross tumor volume (GTV) depicted by a red contour. The red dash line on the bottom right panel depicts the thresholded value of ADC to define the subvolume of low ADC in the gross tumor volume. The mean histograms of the subgroups across tumors were averaged out the individual bimodal distributions, particularly at 2wk. In the individual tumors, single Gaussian-like distributions were observed in 5 tumors pre-RT and 13 tumors at 2wk.

In the LAHNSCC, the patients with NED had the smallest values of mean ADC values in both primary tumor and total nodal tumor volumes pre-RT and at 2wk, of µ_L_ and µ_H_ in primary tumors pre-RT and at 2wk, and of primary and total nodal GTVs pre-RT and at 2wk, compared to the patients with LF (with or without other progression) or DF only, but the differences between the three subgroups were not significant with FDC (p>0.1) (**Supplementary Table 1**). Averaged histograms of pre-RT ADC distributions of primary GTVs associated with NED, LF and DF only pre-RT and 2wk are plotted in **Figure 1**.

**Figure 2:**
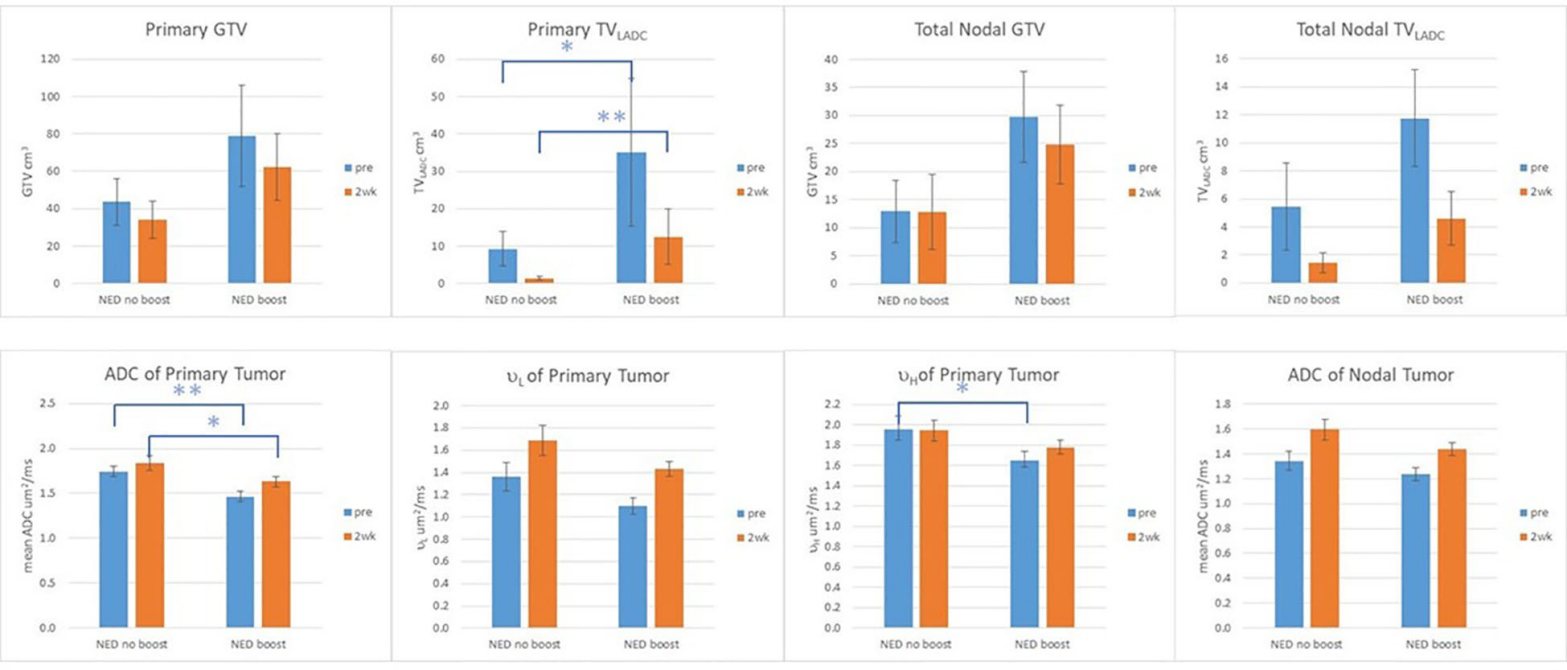
GTV, TV_LADC_, and mean ADC values of primary and nodal tumors, and µ_L_ and µ_H_ of primary tumors pre-RT and at 2wk of the patients with no evidence of disease (NED) with and without boost. Error Bar: standard error of mean; **: p value <0.01: *: p value <0.05.

In the p16+ OPSCC, RT boost effect was observed in FFLRP (**Supplementary Figure 1B**), but also in diffusion metrics (**Supplementary Table 2**). Compared with LF and DF subgroups, the patients with NED_70_ had the greatest mean ADC and the smallest TV_LADC_ in primary tumors pre-RT and at 2wk (p<0.05). The µ_L_ and µ_H_ values of primary tumors followed the same trend as the mean ADC. Compared with the NED_70_ patients, the NED_80_ patients had significantly low values of mean ADC of primary tumors pre-RT and at 2wk and µ_H_ pre-RT (0.004, 0.04, and 0.02, respectively), and had significantly large TV_LADC_ of primary tumor pre-RT and at 2wk (p<0.01, and 0.006, respectively), see **Figure 2**. This suggests that RT boost may overcome higher tumor cellularity or density. **Figure 1** shows the averaged histograms of ADC distributions in primary GTVs of the subgroups pre-RT and 2wk.

### Predictive values of ADC metrics and GTVs for progression

Considering the observed opposite trends in ADC metrics between the p16+ OPSCC and the LAHNSCC, an interaction effect between the diffusion metric and p16 status was suggested. RT boost effect was observed only in the p16+ OPSCC but not in the LAHNSCC, suggesting that RT boost effect interacted with p16 status. To avoid overfitting due to too many co-variables in the multivariate Cox model (e.g., boosting effect, the interactions between boost and the diffusion metrics as well as between boost and p16 status) and to best model the data applied to standard clinical practice, the 21 patients with NED_80_ were excluded from the progression prediction models but 2 patients with LF and with boost were included. All patients with DF were included since there was no boost effect expected for FFDP. In the multivariate Cox models of progression prediction, p16 status, a diffusion metric (TV_LADC_, mean ADC, µ_L_, or µ_H_) and the interaction of the diffusion metric and p16 status were considered.

After adjusting for p16 effect, Cox models for prediction of FFLP found significant effects of pre-RT TV_LADC_, pre-RT mean ADC, and pre-RT µ_L_ of primary tumor for the LAHNSCC (p<0.04 with FDC), and effects of the interactions of pre-RT TV_LADC_, pre-RT mean ADC, 2wk µ_L_ of primary tumor with p16 status (p<0.03 without FDC), but no significant effect of TV_LADC_, mean ADC, µ_L_ or µ_H_ of primary tumor pre-RT or at 2wk for p16+ OPSCC (**Table 2**). After adjusting for p16 effect, Cox models for prediction of FFDP found no significant effects of the tested diffusion metrics of p16+ OPSCC. In LAHNSCC, the significant effects of pre-RT TV_LADC_, pre-RT mean ADC, pre-RT µ_L_ and pre-RT µ_H_ of primary tumor for the LAHNSCC (p<0.04 with FDC), and an effect of the interaction of pre-RT TV_LADC_ of nodal tumor with p16 status (p<0.04 without FDC) were noted (**Table 2**). After adjusting for p16 effect, Cox models for prediction of FFLRDP found significant effects of pre-RT TV_LADC_, pre-RT mean ADC, pre-RT µ_L_ and of pre-RT µ_H_ of primary tumor for the LAHNSCC (p<0.05 with FDC), and significant effects of the interactions of pre-RT TV_LADC_, and pre-RT µ_L_ of primary tumor with p16 status (p<0.02 with FDC), but no significant effect of the tested diffusion metrics of primary or nodal tumor pre-RT or at 2wk for p16+ oropharynx cancers (**Table 3**). After adjusting for p16 status, primary GTV at 2wk predicted significantly for FFLRDF (p<0.03, HR=2.21, GTV > the median value (48.0 cm^3^)) but no primary or nodal GTV pre-RT or 2wk predicted for FFLP or FFDP.

**Table 2:** Multivariate cox models for FFLP and FFDP.

	Variable	FFLP			FFDP		
		HR(95%IC)	P	p w FDC	HR(95%IC)	p	p w FDC
M1	p16 effect	0.19(0.05-0.74)	*0.02^*	0.08	0.25(0.08-0.78)	*0.02^*	0.08
	Pre-RT pTV_LADC_ effect for LAHNC	0.25(0.07-0.88)	*0.03^*	0.04*	0.27(0.09-0.82)	*0.02^*	0.03*
	Pre-RT pTV_LADC_ effect for p16+ OPC	2.20(0.49-9.84)	0.3	0.6	1.44(0.38-5.39)	0.6	0.8
	Difference in pre-RT pTV_LADC_ effects#	8.70(1.24-61.1)	*0.03^*	0.06	5.40(0.93-31.3)	0.06	0.1
M2	p16 effect	1.59(0.40-6.36)	0.5	0.8	1.22(0.34-4.42)	0.8	0.8
	Pre-RT mean pADC effect for LAHNC	3.93(1.13-13.6)	*0.03^*	0.04*	3.71(1.21-11.4)	*0.02^*	0.03*
	Pre-RT mean pADC effect for p16+ OPC	0.48(0.11-2.16)	0.3	0.6	0.81(0.22-3.03)	0.8	0.8
	Difference in pre-RT mean pADC effects#	0.12(0.02-0.86)	*0.03^*	0.06	0.22(0.04-1.22)	0.08	0.1
M3	p16 effect	1.43(0.32-6.39)	0.6	0.8	1.21(0.34-4.25)	0.8	0.8
	Pre-RT u_L_ effect for LAHNC	5.48(1.43-21.1)	*0.01^*	0.04*	4.50(1.41-14.3)	*0.01^*	0.03*
	Pre-RT u_L_ effect for p16+ OPC	0.77(0.17-3.45)	0.7	0.7	0.81(0.22-3.01)	0.8	0.8
	Difference in pre-RT u_1_ effects#	0.14(0.02-1.05)	0.06	0.07	0.18(0.03-1.04)	0.055	0.1
M4	p16 effect	0.97(0.24-3.90)	1.0	1.0	0.86(0.23-3.25)	0.8	0.8
	Pre-RT u_H_ effect for LAHNC	2.70(0.77-9.40)	0.1	0.1	3.34(1.04-10.7)	*0.04^*	0.04*
	Pre-RT u_H_ effect for p16+ OPC	0.76(0.17-3.42)	0.7	0.7	1.32(0.35-4.94)	0.7	0.8
	Difference in pre-RT u_H_ effects#	0.28(0.04-1.96)	0.2	0.2	0.39(0.07-2.23)	0.3	0.4
M5	p16 effect	2.02(0.48-8.54)	0.3	0.6	0.11(0.01-0.86)	*0.04^*	0.16
	2wk u_L_ effect for LAHNC	4.08(1.07-15.5)	*0.04^*	0.2	0.57(0.18-1.88)	0.4	1.0
	2wk u_L_ effect for p16+ OPC	0.30(0.06-1.54)	0.15	0.3	7.12(0.89-57.1)	0.06	0.24
	Difference in pre-RT u_L_ effects#	0.07(0.01-0.61)	*0.02^*	0.08	12.4(1.1-136.0)	*0.04^*	0.16
M6	p16 effect	0.89(0.24-3.32)	0.9	0.9	0.51(0.13-1.89)	0.3	0.3
	2wk u_H_ effect for LAHNC	2.27(0.69-7.52)	0.2	0.2	1.02(0.29-3.63)	1.0	1.0
	2wk u_H_ effect for p16+ OPC	0.61(0.14-2.74)	0.5	0.5	0.91(0.22-3.84)	0.9	0.9
	Difference in pre-RT u_2_ effects#	0.27(0.04-1.84)	0.2	0.2	0.97(0.14-6.60)	1.0	1.0
M7	p16 effect	0.60(0.22-1.60)	0.3		1.25(0.36-4.38)	0.7	0.9
	Pre PGTV	1.52(0.57-3.99)	0.4		2.74(0.91-8.23)	0.07	0.1
M8	p16 effect	0.60(0.22-1.58)	0.3		0.60(0.16-2.25)	0.5	0.8
	2wk PGTV	1.87(0.70-4.98)	0.2		0.22(0.04-1.24)	0.09	0.4

LAHNC, locoregionally advanced head and neck squamous cell carcinoma; p16+ OPC, p16+ oropharynx cancer; # difference in the parameter effects between p16+ OPC and LAHNC (the interaction of the parameter with p16 status). HR for TV_LADC_ of primary tumor pre-RT > the median value (15.3 cm^3^); HR for mean ADC of primary tumor pre-RT > the median value (1.48 um^2^/ms); HR for u_L_ of primary tumor pre-RT > the median value (1.08 um^2^/ms); HR for u_H_ of primary tumor pre-RT > the median value (1.67 um^2^/ms). Tested models are marked as M1-8.

P value with false discovery control (FDC) < 0.05 is considered as significance and marked in bold and by *.

**Table 3:** Multivariate cox models for FFLRDP.

Model	Variable	FFLRDP		
		HR(95%IC)	p	P w FDC
M1	p16 effect	0.32(0.12-0.86)	*0.02^*	0.08
	Pre-RT pTV_LADC_ effect for LAHNC	0.37(0.14-0.98)	*0.04^*	0.05*
	Pre-RT pTV_LADC_ effect for p16+ OPC	2.21(0.82-5.95)	0.1	0.4
	Difference in pre-RT pTV_LADC_ effects#	5.93(1.47-23.9)	*0.01^*	0.02*
M2	p16 effect	1.54(0.59-4.04)	0.4	0.5
	Pre-RT mean pADC effect for LAHNC	2.64(1.01-6.87)	*0.05^*	0.05*
	Pre-RT mean pADC effect for p16+ OPC	0.66(0.25-1.75)	0.4	0.8
	Difference in pre-RT mean pADC effects#	0.25(0.06-0.97)	*0.05^*	0.07
M3	p16 effect	1.74(0.61-4.94)	0.3	0.5
	Pre-RT u_L_ effect for LAHNC	4.86(1.75-13.5)	*0.002^*	0.008*
	Pre-RT u_L_ effect for p16+ OPC	0.81(0.30-2.18)	0.7	0.9
	Difference in pre-RT u_1_ effects#	0.17(0.04-0.69)	*0.01^*	0.02*
M4	p16 effect	1.19(0.43-3.29)	0.7	0.7
	Pre-RT u_H_ effect for LAHNC	2.81(1.05-7.55)	*0.04*^	0.05*
	Pre-RT u_H_ effect for p16+ OPC	1.06(0.40-2.84)	0.9	0.9
	Difference in pre-RT u_H_ effects#	0.38(0.10-1.49)	0.2	0.2
M5	p16 effect	0.39(0.12-1.24)	0.1	0.4
	Pre-RT nTV_LADC_ effect for LAHNC	0.90(0.34-2.38)	0.8	0.8
	Pre-RT nTV_LADC_ effect for p16+ OPC	2.71(0.87-8.44)	0.09	0.3
	Difference in pre-RT nTV_LADC_ effects#	3.01(0.68-13.4)	0.1	0.4
M6	p16 effect	0.61(0.19-1.93)	0.4	0.4
	Pre-RT mean nADC effect for LAHNC	1.13(0.37-3.48)	0.8	0.8
	Pre-RT mean nADC effect for p16+ OPC	1.79(0.64-5.00)	0.3	0.3
	Difference in pre-RT mean nADC effects#	1.59(0.35-7.20)	0.5	0.5
M7	p16 effect	1.80(0.67-4.76)	0.2	0.3
	2wk u_L_ effect for LAHNC	2.46(0.95-6.39)	0.06	0.2
	2wk u_L_ effect for p16+ OPC	0.45(0.16-1.24)	0.1	0.2
	Difference in pre-RT u_L_ effects#	0.18(0.05-0.74)	*0.02^*	0.08
M8	p16 effect	1.03(0.38-2.77)	0.9	0.9
	2wk u_H_ effect for LAHNC	2.20(0.85-5.71)	0.1	0.2
	2wk u_H_ effect for p16+ OPC	1.09(0.40-2.96)	0.9	0.9
	Difference in pre-RT u_H_ effects#	0.52(0.13-2.02)	0.3	0.3
M9	p16 effect	0.94(0.46-1.91)	0.9	
	Pre PGTV	2.00(0.98-4.10)	0.06	
M10	p16 effect	0.90(0.45-1.83)	0.8	
	2wk PGTV	2.21(1.08-4.56)	**0.03**	

LAHNC, locoregionally advanced head and neck squamous cell carcinoma; p16+ OPC, p16+ oropharynx cancer; # difference in the parameter effects between p16+ OPC and LAHNC (the interaction of the parameter with p16 status). HR for TV_LADC_ of primary tumor pre-RT > the median value (15.3 cm^3^); HR for mean ADC of primary tumor pre-RT > the median value (1.48 um^2^/ms); HR for u_L_ of primary tumor pre-RT > the median value (1.08 um^2^/ms); HR for u_H_ of primary tumor pre-RT > the median value (1.67 um^2^/ms). Tested models are marked as M1-10.

P value with false discovery control (FDC) < 0.05 is considered as significance and marked in bold and by *.

**Figure 3:**
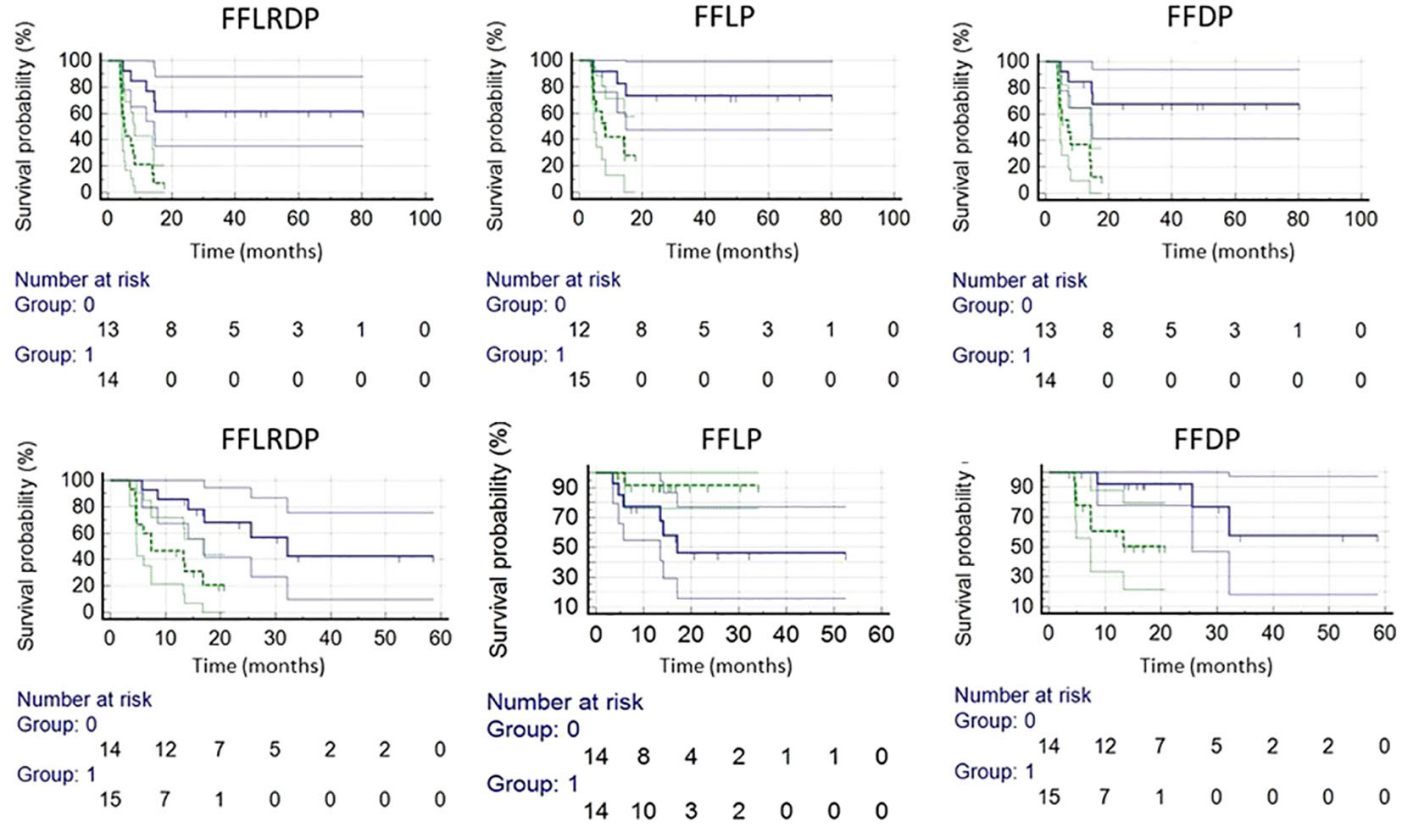
Kaplan-Meier plots of LAHNSCC (top row) and p16+ OPSCC (bottom row). LAHNSCC from left to right: freedom from locoregional and distant progression (FFLRDP) predicted by the µ_L_ value of primary tumor pre-RT(p<0.0003), and freedom from local progression (FFLP) predicted by the µ_L_ value of primary tumor pre-RT (p<0.004), and freedom from distant progression (FFDP) predicted by the µ_L_ of primary tumor pre-RT (p<0.002). p16+ OPSCC from left to right: FFLRDP predicted by primary GTV pre-RT (p<0.005), FFLP predicted by the mean ADC value of primary tumor pre-RT (p<0.06), and FFDP by primary GTV pre-RT (p<0.02). Blue and green lines are for respective small and large values of the tested metrics split by the median values.

Post *ad hoc* analysis confined to patients with p16+ OPSCC showed that only TV_LADC_ of the total nodal burden pre-RT was a significant predictor for FFDP (p=0.05, HR=8.02(0.99-65.2) for nodal TV_LADC_ >2.3 cm^3^), and primary GTVs pre-RT and at 2wk were significantly predictors for FFLRDF (p<0.02, HR=3.52(1.21-10.2) for GTV pre-RT > 57.1 cm^3^ and p<0.02, HR=3.90(1.30-11.7) GTV 2wk >48.0 cm^3^). Kaplan-Meier curves of FFLP, FFDP and FFLDP in the p16+ OPSCC and LAHNSCC are shown in **Figure 3**.

## Discussion

In this study, we assessed quantitative diffusion metrics and ADC histograms for their associations with tumor progression in the patients with locally advanced p16+ OPSCC and LAHNSCC (95% of T4/N3) and enrolled on a randomized phase II trial of adaptive RT boost. With expected outcome differences between the advanced p16+ OPSCC and LAHNSCC, we found different ADC characteristics and associations with tumor progression between the two. Particularly, for the LAHNSCC, low ADC and large TV_LADC_ were associated with low risk of local and distant tumor progression, which could be interpreted as a lower ADC value associated with a less extent of stroma in tumor. However, for the p16+ OPSCC, high ADC, and small TV_LADC_ were associated with a trend of low risks of local and distant tumor progression, which may be attributed to tumor infiltrating lymphocytes. In analysis confined to p16+ OPSCC, we did see that nodal TV_LADC_ may be an imaging marker for distant progression, and primary GTV pre-RT and at 2 weeks during RT seem to be a stronger predictor for local, regional and distant progression albeit with the limitations of *ad hoc* analysis. As all, the ADC value appears to be affected substantially by biology and morphology of p16- and p16+ tumors as well as associated heterogeneity. Diffusion images, although easily acquired, could be affected by many biological, clinical and physical factors. Further investigations of these factor effects on diffusion images through pathologic correlation are needed to guide either radiation de-escalation or treatment intensification trials using ADC metrics.

ADC is sensitive to tumor microstructure. Histologically, p16- HNSCC is typically comprised of a keratinizing morphology with angulated nests of tumor cells, abundant cytoplasm, stromal desmoplasia, and central necrosis; p16+ oropharynx cancer is typically described as a nonkeratinizing morphology with the presence of a large amount of tumor infiltrating lymphocytes as well as small amounts of cytoplasm, central necrosis and stroma (25). These differing morphological features affect the ADC value and the ADC distribution in these tumors. For instance, it has been reported that the ADC value is positively correlated to the total percentage area of stroma and inversely correlated to the cell density in the HN tumors (26). When the tumor morphology variation manifests in the macroscopic level, i.e., in the millimeter range, an effect can be observed in the ADC value and distribution. It is plausible that in the p16- tumors, a low mean ADC value or low peak ADC values of the two components indicate a low total percentage of stroma, and thereby less protection of tumor cells from CRT by stroma and better tumor control (27–32). In the p16+ oropharynx tumors, tumor microstructure may be affected by tumor infiltrating lymphocytes (33, 34), where a large subvolume with low ADC in the tumor volume, low mean ADC or low µ_L_ value, could need to be treated with high radiation doses to have an improved local and regional tumor control. ADC is measured at the macroscopic level but affected by very different microscopic morphology and biology. All these challenge the analysis and interpretation of ADC and ADC changes in HNSCC.

It is not entirely clear how p16 status affects tumor ADC distribution changes in response to CRT. Although after 20 Gy of radiation both types of tumors show an increase in mean ADC values, the LAHNSCC tumors maintained the bi-distribution in a certain extent while the p16+ oropharynx tumors show a rapid normalization of the ADC distributions. Note that the histograms in **Figure 1** are from the subgroup averages, which further remove individual variations. Nevertheless, a mean ADC in the tumor could over-simplify the heterogeneity of ADC distribution in HNSCC, particularly for the locally advanced tumors, and could obscure the different responses between p16- and p16+ tumors. A histogram analysis of the ADC bi-distribution or the low ADC component (e.g., subvolumes of low ADC defined by a threshold) could remove confounding effects to an extent and thereby increase the predictive power for progression. While ADC metrics in HNSCC are useful to differentiate tumor control vs progression, primary GTV in the p16+ oropharynx tumors seems to have sufficient power for prediction of local, regional and distant progression, but not in other LAHNSCC tumors.

This study has a few limitations. The number of patients in this study is still small. In line with standard clinical practice, our LAHNSCC patients included p16- oropharynx cancer as well as both p16+ and p16- non-oropharyngeal cancers. The potential biologic or clinical significance of p16 status outside the oropharynx is unclear with some suggestion that p16 positivity outside the oropharynx is less correlated with HPV positivity and may be driven by other molecular mechanisms (12, 13). However, the tumor ADC distribution could be different at different sites. The 21 p16+ oropharynx patients who received 80 Gy local RT boost and had no progression had large GTVs and ADC metrics for high risk of tumor progression. To avoid overfitting, the 21 patients were excluded from the progression analysis. Nevertheless, characterizing the ADC metrics in these two groups of patients according to current clinical practice further reveals differences between the two types of HNSCC, and has the potential to improve the power of the ADC metrics as a biomarker for assessment of tumor response and prediction of progression in HNSCC. Considering complexity of tumor biology and response to chemoradiation therapy, integrating multi-imaging biomarkers, including FDG PET and dynamic contrast enhanced MRI, as well as liquid biomarkers (e.g., circulating tumor human papilloma virus DNA) during the early course of CRT could improve prediction of tumor progression, which could provide sufficient time and guidance for individualized (intensified or de-intensified) adaptation of CRT and thereby improve outcomes of patients with locally advanced head and neck cancers.

## Data availability statement

The raw data supporting the conclusions of this article will be made available by request to the corresponding author.

## Ethics statement

The studies involving humans were approved by University of Michigan Review Board. The studies were conducted in accordance with the local legislation and institutional requirements. The participants provided their written informed consent to participate in this study.

## Author contributions

YC: study design, oversight, data analysis, manuscript writing. MA: image analysis. PL: statistical modeling. CL: support data collection. MS: statistical modeling. DY: software support for image analysis. EJ: patient recruitment and clinical data collection. LG: patient recruitment and clinical data collection. JS: patient recruitment and clinical data collection. AE: study design and patient recruitment. MM: patient recruitment, clinical data collection and manuscript writing. All authors contributed to the article and approved the submitted version.
