# Supplementary figures and images for: Diffusion MRI correlation with p16 status and prediction for tumor progression in locally advanced head and neck cancer
Source: Front Oncol. 2023 Dec 21;13:998186. doi: 10.3389/fonc.2023.998186 (PMC10771284; doi:10.3389/fonc.2023.998186)

Supplementary Figure 1


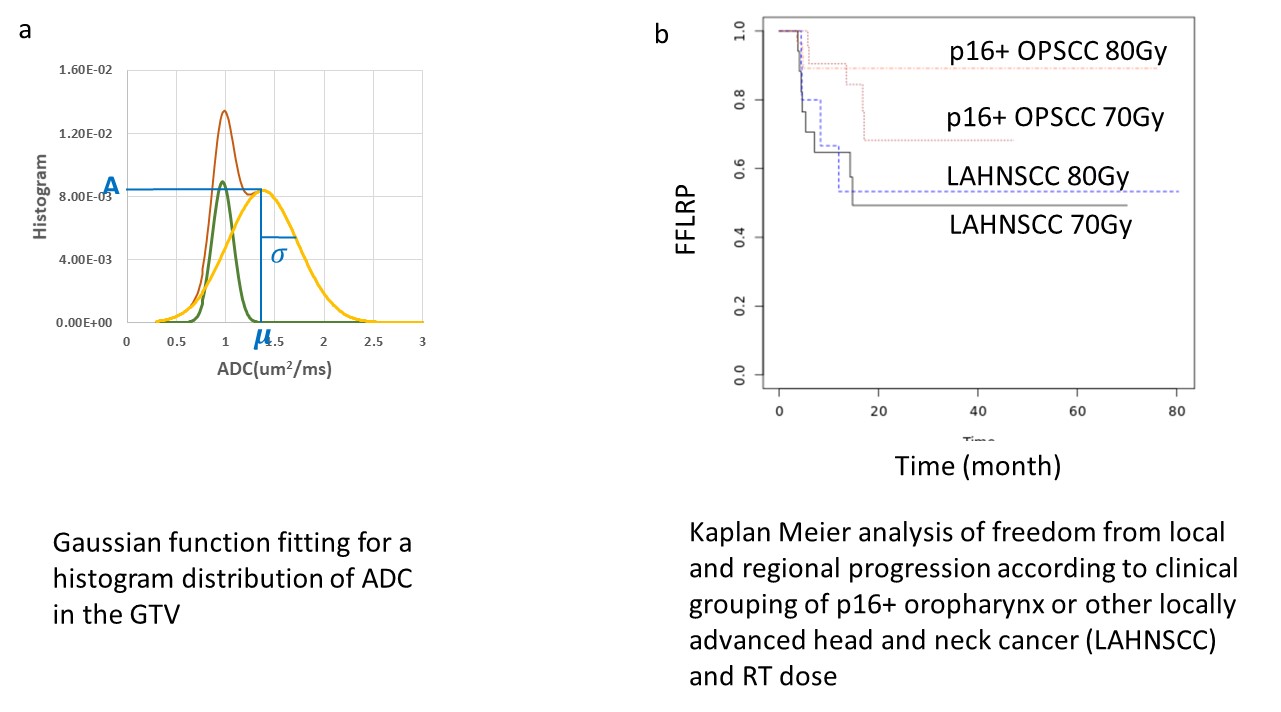

Supplement: Supplementary file 1 [file DataSheet_1.docx]

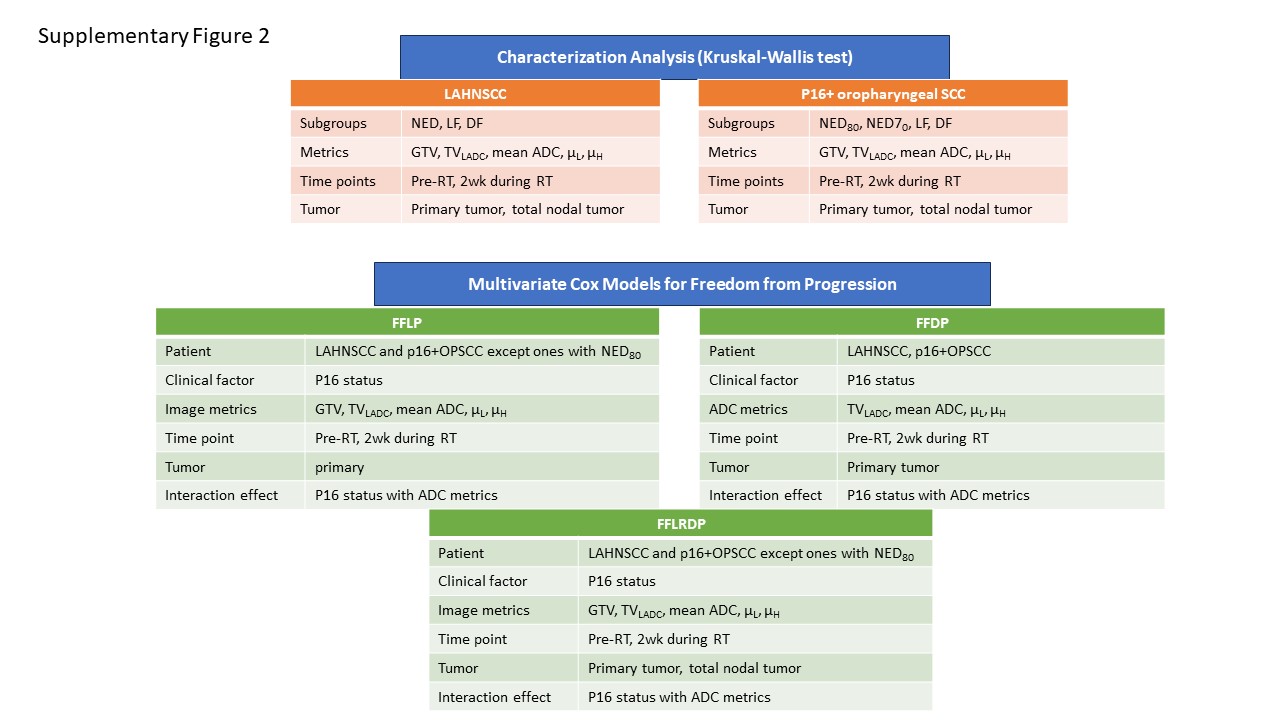

Supplement: Supplementary file 2 [file Image_2.jpeg]
