# Supplementary material for: Diffusion MRI correlation with p16 status and prediction for tumor progression in locally advanced head and neck cancer
Source: Front Oncol. 2023 Dec 21;13:998186. doi: 10.3389/fonc.2023.998186 (PMC10771284; doi:10.3389/fonc.2023.998186)
Supplement: Supplementary file 3 [file Table_1.docx]

Supplementary Table 1. ADC metrics and GTV in LAHNSCC patients

| LAHNSCC | Primary Tumor  pre-RT (mean(sd)) | | Primary Tumor  2wk (mean(sd)) | | Total Nodal Tumor  pre-RT (mean(sd)) | | Total Nodal Tumor  2wk (mean(sd)) | |
| --- | --- | --- | --- | --- | --- | --- | --- | --- |
|  | TV_LADC_ | ADC | TV_LADC_ | ADC | TV_LADC_ | ADC | TV_LADC_ | ADC |
| NED | 24.8(11.7) | 1.32(0.21) | 8.3(5.1) | 1.53(0.16) | 14.1(27.0) | 1.25(0.15) | 9.4(18.1) | 1.29(0.17) |
| LF | 28.0(45.2) | 1.56(0.31) | 11.4(19.8) | 1.78(0.28) | 25.2(44.1) | 1.37(0.40) | 8.7(15.1) | 1.41(0.36) |
| DF | 24.0(15.1) | 1.46(0.30) | 11.3(6.2) | 1.68(0.31) | 13.0(24.4) | 1.34(0.29) | 5.7(13.8) | 1.53(0.25) |
| KW p | 0.5 | 0.15 | 0.4 | 0.1 | 0.5 | 0.4 | 1.0 | 0.09 |
| p w FDC | NS | NS | NS | NS | NS | NS | NS | NS |
|  | µ_L_ | µ_H_ | µ_L_ | µ_H_ |  |  |  |  |
| NED | 0.97(0.20) | 1.47(0.35) | 1.15(0.14) | 1.60(0.15) |  |  |  |  |
| LF | 1.28(0.32) | 1.84(0.40) | 1.39(0.26) | 1.97(0.41) |  |  |  |  |
| DF | 1.07(0.20) | 1.75(0.42) | 1.32(0.34) | 1.85(0.43) |  |  |  |  |
| KW | *0.02^* | 0.07 | 0.3 | 0.08 |  |  |  |  |
| p w FDC | 0.1 | 0.2 | 0.5 | 0.2 |  |  |  |  |
|  | GTV |  | GTV |  | GTV |  | GTV |  |
| NED | 58.0(20.2) |  | 50.9(27.7) |  | 31.5(57.8) |  | 28.8(50.1) |  |
| LF | 93.0(72.6) |  | 70.0(41.0) |  | 89.5(97.2) |  | 43.7(70.2) |  |
| DF | 70.3(28.7) |  | 62.8(33.7) |  | 43.4(81.8) |  | 36.1(61.2) |  |
| KW p | 0.5 |  | 0.4 |  | 0.6 |  | 0.6 |  |
| p w FDC | NS |  | NS |  | NS |  | NS |  |

LAHNSCC includes p16- oropharynx cancer as well as p16+/p16- non-oropharyngeal sites; TV_LADC_ : subvolume of the tumor with LADC < 1.2 um^2^/ms; ADC: mean value of ADC in the GTV; NED: no evidence of disease *: p value with FDC < 0.05, significant; ^: p value without FDC < 0.05. TV_LADC_ and GTV are in unit of cm^3^. ADC, µ_L_ and µ_H_ are in unit of um^2^/ms. 10 patients had NED, 11 had primary tumors had LF with or without RF or DF, and 7 had DF only, which were used to stratify primary tumors. For nodal tumors, the 14 patients with DF with or without LRF were grouped, and 4 with LF without DF were grouped. There was no patient who had RF only.
