# Supplementary material for: Diffusion MRI correlation with p16 status and prediction for tumor progression in locally advanced head and neck cancer
Source: Front Oncol. 2023 Dec 21;13:998186. doi: 10.3389/fonc.2023.998186 (PMC10771284; doi:10.3389/fonc.2023.998186)
Supplement: Supplementary file 4 [file Table_2.docx]

Supplementary Table 2. ADC metrics and GTV as predictors of progression in p16+ oropharynx cancer patients

| p16+ | Primary Tumor  pre-RT (mean(sd)) | | Primary Tumor  2wk (mean(sd)) | | Total Nodal Tumor  pre-RT (mean(sd)) | | Total Nodal Tumor  2wk (mean(sd)) | |
| --- | --- | --- | --- | --- | --- | --- | --- | --- |
|  | TV_LADC_ | ADC | TV_LADC_ | ADC | TV_LADC_ | ADC | TV_LADC_ | ADC |
| NED_80_ | 35.1(83.5) | 1.47(0.26) | 12.5(32.4) | 1.63(0.24) | 11.8(15.4) | 1.24(0.21) | 4.6(8.3) | 1.44(0.21) |
| NED_70_ | 9.3(14.6) | 1.74(0.18) | 1.4(1.5) | 1.83(0.25) | 5.4(10.3) | 1.34(0.21) | 1.4(2.1) | 1.60(0.23) |
| LF | 18.9(10.1) | 1.45(0.17) | 9.9(7.1) | 1.56(0.18) | 10.6(12.4) | 1.37(0.23) | 6.0(11.2) | 1.59(0.19) |
| DF | 15.5(6.7) | 1.45(0.18) | 6.3(6.2) | 1.66(0.18) | 13.8(13.5) | 1.27(0.25) | 7.2(9.7) | 1.50(0.24) |
| KW p | *0.03^* | *0.006^* | *0.01^* | *0.05^* | *0.05^* | 0.7 | 0.2 | 0.7 |
| p w FDC | ***0.05**** | ***0.02**** | ***0.05**** | ***0.05**** | 0.2 | 0.7 | 0.3 | 0.7 |
|  | µ_L_ | µ_H_ | µ_L_ | µ_H_ |  | |  | |
| NED_80_ | 1.04(0.27) | 1.65(0.41) | 1.38(0.28) | 1.81(0.30) |  |  |  |  |
| NED_70_ | 1.28(0.37) | 2.04(0.37) | 1.47(0.26) | 2.05(0.31) |  |  |  |  |
| LF | 1.00(0.15) | 1.68(0.37) | 1.12(0.15) | 1.72(0.21) |  |  |  |  |
| DF | 0.98(0.19) | 1.64(0.23) | 1.33(0.31) | 1.81(0.22) |  |  |  |  |
| KW p | 0.1 | *0.03^* | *0.04^* | 0.07 |  |  |  |  |
| p w FDC | 0.2 | 0.1 | 0.1 | 0.1 |  |  |  |  |
|  | GTV |  | GTV |  | GTV |  | GTV |  |
| NED_80_ | 79.1(123.5) |  | 62.4(81.1) |  | 29.7(37.1) |  | 24.8(32.0) |  |
| NED_70_ | 43.6(43.3) |  | 34.0(34.4) |  | 12.9(19.2) |  | 12.8(23.4) |  |
| LF | 59.7(36.3) |  | 52.6(32.2) |  | 28.6(26.7) |  | 25.1(26.8) |  |
| DF | 53.3(21.1) |  | 46.0(19.3) |  | 29.0(19.5) |  | 27.9(19.7) |  |
| KW p | 0.2 |  | 0.1 |  | *0.05^* |  | 0.06 |  |
| p w FDC | 0.2 |  | 0.1 |  | 0.1 |  | 0.1 |  |

TV_LADC_ : subvolume of the tumor with LADC < 1.2 um^2^/ms; ADC: mean value of ADC in the GTV; NED_70_: no evidence of disease in the 70 Gy arm; NED_80_: no evidence of disease in the 80 Gy arm. *: p value with FDC < 0.05, significant; ^: p value without FDC < 0.05. TV_LADC_ and GTV are in unit of cm^3^. ADC, µ_L_ and µ_H_ are in unit of um^2^/ms. KW: Kruskal-Wallis test. 34 patients had NED with 13 treated at 70 Gy (NED_70_) and 21 NED treated at 80 Gy (NED_80_), 7 had LF without DF, and 9 had DF without LF, which were used to stratify primary and nodal tumors. Boosting was not expected to affect distant progression. For the 7 patients with LF, in which 5 had no boost and 2 had boost, due to the small number of the patients with LF, the 7 patients were not divided in this Table.
